# In Memory of Professor John Gordon McVie: Bringing People Together 13 January 1945 – 20 January 2021

**DOI:** 10.3389/fonc.2021.664035

**Published:** 2021-02-25

**Authors:** Giuseppe Giaccone

**Affiliations:** Weill-Cornell Medicine, New York, NY, United States

**Keywords:** in memoriam, in memory, obituaries, biographies, John Gordon McVie

Gordon has been a mentor for me, and one of my best friends for 40 years. What has always impressed me of Gordon was his innate ability to bring people to work together, to build new structures and to facilitate cancer research in creative new ways. Those who knew him will remember his sharp thinking, his wit and great sense of humor and his upbeat spirit and love for life, work and fun. I will never forget his kindness, his balanced advice, his jokes, and his incredibly fast speaking.

Professor Gordon McVie qualified in the nineteen sixties in science and medicine at Edinburgh University, he was appointed Foundation Senior Lecturer at the Cancer Research Campaign oncology unit at the University of Glasgow in 1975. He trained in the US, and spent sabbaticals in Paris, Sydney, and Amsterdam.

Throughout the eighties, he was Clinical Research Director at the Netherlands Cancer Institute in Amsterdam, and set up their drug discovery programme and the leading intraperitoneal chemotherapy studies in Europe. In the early eighties he was the Chair of the European Organization for Research and Treatment of Cancer (EORTC) Lung Cancer Cooperative Group for 6 years. I met him for the first time in Brussels at one of the first meetings of this newly formed group. He was also appointed Chair of the UICC Fellowships Programme in 1990 for 8 years. From 1994 to 1997 he served as President of the EORTC, and during that time he set up the present Drug Development Group in Brussels, and with the US National Cancer Institute support, the European New Drug Development Network.

After almost a decade in the Netherlands, Professor McVie returned to the UK to be the Chief Executive of the Cancer Research Campaign (CRC), which, under his aegis, took over 70 molecules from the lab into clinical trial. He led CRC into a merger with Imperial Cancer Research Fund in 2002, which formed the largest cancer charity in the UK, Cancer Research UK. In the UK he was one of the architects of the Cancer Trials Networks in Scotland, Wales, and England, and was a founding member of the National Cancer Research Institute. His commitment to drug discovery and delivery is evidenced by ~240 patents granted to CRC scientists under his leadership, several drugs registered including carboplatin, temozolomide, olaparib, and abiraterone and the foundation of 10 biotechnology companies based on CRC intellectual property. His clinical interests, apart from new drug discovery and chemoprevention were in the management of cancers of the lung, ovary, liver, breast, and brain.

Lately he was visiting professor at Cancer Studies, Kings College London for five years, and was Clinical Research Advisor to the Institute of Molecular Oncology (IFOM) in Milan. He was founding editor of ecancer.org and ecancerpatient.org both online Open Access free websites, with almost 21 million visits in a decade from 191 countries. For 10 years he was Senior Consultant building clinical research at the European Institute of Oncology in Milan.

He was a Director and Co-Founder of Ellipses Pharma - an advanced cancer medicines company.

Professor McVie was the recipient of numerous awards and has honorary doctorates in science and medicine from six universities. He has served on key committees of AACR and ASCO, and on the boards of the National Cancer Institutes of France, Italy, and Holland. He has authored 360 peer-reviewed articles, and contributed to over 35 books.

He was elected as Fellow of the European Association for Cancer Science in 2014, and has been chairman of the European Alliance for Personalized Medicine since early 2016.

This is only a short selection of the most important activities Prof. McVie has been engaged in. He was truly a dynamic international presence that recognized the necessity of going beyond the geographical boundaries, bringing people together to create novel structures in the pursuit of the fight against cancer.

## Author Contributions

The author confirms being the sole contributor of this work and has approved it for publication.

## Conflict of Interest

The author declares that the research was conducted in the absence of any commercial or financial relationships that could be construed as a potential conflict of interest.

